# Concussions: A Review of Physiological Changes and Long-Term Sequelae

**DOI:** 10.7759/cureus.54375

**Published:** 2024-02-17

**Authors:** Hemangi Patel, Sneha Polam, Roody Joseph

**Affiliations:** 1 Sports Medicine, Nova Southeastern University Dr. Kiran C. Patel College of Osteopathic Medicine, Fort Lauderdale, USA

**Keywords:** sports injury, physiology, athlete, sports, sequelae, concussion

## Abstract

A concussion is a type of mild traumatic brain injury (mTBI). It is prevalent among athletes across a wide variety of sports. The exact mechanism of a concussion is unknown, but it is currently accepted that the acceleration and deceleration of the brain is the insult causing disturbances in activity. The most common symptoms of concussions include but are not limited to dizziness, nausea, vomiting, and headaches. With repetitive concussive injuries, athletes can experience permanent changes such as chronic traumatic encephalopathy (gradual degeneration of brain tissue), which can lead to personality changes and memory deficits. This literature review aims to provide insight into concussions and the evaluation of physiological changes and long-term sequelae. A comprehensive literature search was performed from April 2012 to April 2022 using PubMed/MEDLINE (Medical Literature Analysis and Retrieval System Online) and Embase databases. Nineteen articles were finally included in the literature review. The review shows that neuroimaging results demonstrated significant changes in the brain structure and function including alternations in the thalamus, hippocampus, corpus callosum, and the white matter, which can extend beyond the symptom amelioration. In addition, other approaches include brain metabolism, cerebral blood flow, and glucose utilization. Additional techniques include the evaluation of fatigue levels and the alterations in biomarkers, specifically IL-6. These approaches have demonstrated that consequences of concussions, including alternations in brain structure and function, can extend beyond an athlete’s report of symptom resolution, and should be taken into consideration for return to play. The physiological changes present after a concussion have the potential to develop into long-term complications such as academic difficulty, cognitive decline, gut microbiome changes, gait changes, and increased risk of lower extremity injury. The findings in the literature review determine that is important to immediately address the symptoms of concussions to expedite treatment and prevent deleterious changes to the brain.

## Introduction and background

A concussion is categorized as a mild traumatic brain (mTBI) injury that involves an injury to the head, with or without loss of consciousness. Although a complete understanding of concussions has yet to be fully elucidated, evidence proposes that the underlying mechanism involves the brain undergoing rotational acceleration and deceleration [1]. This movement can result in tearing of axons and a disruption in the brain’s metabolic glucose cascade [2]. This brain injury can result in a range of lasting complications such as cognitive impairments, altered physical function, and lack of emotional regulation [3]. Frequent indicators of concussions include feelings of lightheadedness, headaches, nausea, and vomiting [4]. While these injuries can present with severe and incapacitating symptoms, conventional neuroimaging methods typically do not reveal any anomalies. Furthermore, many athletes report improvement in their symptoms and a return to baseline within a week of sustaining the injury. Consequently, concussions have been characterized as inconsequential or minor injuries lacking lasting repercussions, and athletes are frequently permitted to return to play shortly after their symptoms have resolved [5].

Individuals who frequently participate in contact sports can face a higher likelihood of sustaining a sport-related concussion. A history of repeated sport-related concussions can result in debilitating and significant long-term consequences [6]. Recently, advanced neuroimaging techniques have been used to evaluate changes in the structure and function of the brain [5]. These techniques, which encompass assessing brain metabolism, cerebral blood flow, and glucose utilization, imply that alterations in brain function can persist beyond an athlete’s report of symptom resolution [5]. Diffusion tensor imaging (DTI) is a novel technique that assesses water diffusion in the brain tissue, with a focus on white matter tracts. DTI examines fractional anisotropy (FA) and mean diffusivity (MD), markers of white matter damage, which can be modified in patients with TBI and concussed brains. Examining the changes in water diffusion can aid in developing an understanding of the extent of brain damage and create an approach for treatment and rehabilitation [3].

Studies have shown that even after the symptoms of a concussion have resolved, changes to brain function can persist. Through advanced neuroimaging techniques, researchers have found that alterations in the brain may continue even if the athlete appears to be symptom-free [5]. Long-term effects of unresolved concussions can manifest as slower recovery times and can lead to further injuries. They can also cause difficulty in academic performance and have been associated with depression and other mental health complications [7,8].

This review aims to provide insight into the chemical and structural changes in the brain structure, function (including cognition), and long-term physical sequelae following a concussion or multiple concussive events.

## Review

Study design

A comprehensive literature search was performed from April 2012 to April 2022 using PubMed/MEDLINE (Medical Literature Analysis and Retrieval System Online) and Embase databases. The inclusion criteria sought studies that were peer-reviewed, written in English, and performed on human subjects. Exclusion criteria involved non-English studies, animal studies, and literature reviews. Key terms used in the search included "concussion", "effects", "recovery", "therapeutics", "OMM", "osteopathic manipulative medicine", "athletes", "sports" and "treatment". The initial search resulted in 163 articles. This was followed by a second-tier review that focused on articles involving athletes with diagnosed concussions and treatment approaches, resulting in a final count of 19 articles included in the literature review.

Neurological changes due to concussions

Evaluation of Brain Changes Through Neuroimaging

To determine the structural and chemical alterations in the brain due to concussions, researchers have evaluated neuroimaging techniques to assess the potential for changes. Concussions have been shown to cause a change in brain function without the presence of any obvious bleeding or lesions and can lead to long-term consequences [3]. Neuroimaging techniques allow researchers to non-invasively examine the brain's anatomy, connectivity, and function, and provide valuable insights into the underlying mechanisms of brain changes. DTI is a technique that assesses water diffusion in the brain tissue, with a specific focus on white matter tracts like the corpus callosum [3]. The corpus callosum plays a central role in concussions, which is why it is a focus of study, as it serves to navigate communication between the left and right sides of the brain. By analyzing changes in water diffusion, researchers can gain a better understanding of the extent and severity of brain damage, and develop effective strategies for diagnosis, treatment, and rehabilitation. DTI examines the markers of white matter damage, FA, and MD, which can be altered in patients with TBI, and concussed brains. An increase in FA and a decrease in MD may contribute to changes in neuronal growth patterns [3], which have been shown to be associated with cognitive dysfunction [6]. An increase in FA has been shown to be tied to hindered water diffusion, regrowth of axons, and scarring within the white matter. In addition, a decrease in MD relates to correlation in the presence of injury [3]. DTI is specifically focused on water diffusion, which can be limited due to the complexity of the brain structure [3]. Swelling that occurs during a concussion can also impact the diffusion of water and potentially alter the results of the study.

The limitation of DTI in assessing concussions can be addressed by using Diffusion Kurtosis Tensor Imaging (DKTI), a neuroimaging technique that is sensitive to the subacute and chronic phases of the injury [5]. DKTI also has been shown to indicate an increase in FA and a decrease in MD, similar to DTI. Notably, when assessing white matter disruption at the 24-hour mark compared to one week following a sports-related concussion, there has been a greater degree of disruption observed at the one-week mark using DKTI. This shows there is an evolving change to the white matter structures of the brain as time goes on after injury, which can then cause long-term changes [5]. There was no correlation seen between cognitive performance and white matter changes, which differs from the above study that focused on the DTI technique assessing water diffusion in brain tissue. In addition, the absence of significant differences in symptoms between the concussed and normal groups at the day-8 mark indicates that most symptoms may decrease after one week of injury [5]. Meanwhile, research efforts have demonstrated that white matter changes continued to evolve, emphasizing the need for further studies to be conducted to comprehensively understand the recovery timeline of athletes after experiencing a concussion [5].

Cortical Thinning and Thickening

Another mechanism to identify changes in concussion patients is cortical thickness [9]. Studies have demonstrated the relationship between increased levels of trauma and decreased cortical thickening among Veterans with evidence of cortical atrophy after a mTBI [9]. In sports-related mTBI or concussions, cortical thinning has been shown to be present in the prefrontal cortex region and the right inferior parietal cortex [9]. Atorvastatin, a medication that has the potential to increase cortical thickness by reducing inflammation in the brain and improving blood flow, has been studied for its effects on brain atrophy. However, the use of this medication demonstrated no difference in the level of cortical thickening [9]. It would be beneficial to perform a longitudinal study with a larger sample size to determine if sex and age play a role in the presence of cortical thinning in different regions of the brain and the impacts of using a medication that promotes cortical thickening.

IL-6 Elevation, MiR-27a-3p and MiR-221-3p, and Increased Biomarkers

Concussions are diagnosed based on the provider's physical evaluation. Clinical diagnosis of a concussion using biomarkers or unique biosignatures is an area of ongoing research. Biomarkers or unique biosignatures may be used in monitoring the progression of concussions and assessing the effectiveness of treatment methods employed, which may serve as a diagnostic tool as many biomarkers are linked to brain injury, cell damage, and inflammation [10].

Inflammation has been shown to be associated with concussive insult, with elevated inflammatory markers including IL-6 and IL-1. Inflammation can be detected via blood samples with elevated cytokine levels, interleukin levels, and tumor necrosis factor (TNF) within 24 hours after injury and has a correlation with worsened results from the Glasgow Outcome Score [11]. IL-10, IL-6 (which is associated with duration of symptoms and recovery), and c-reactive protein (CRP) have been demonstrated to be elevated six hours after a mTBI (when inflammatory markers and symptom severity are at their peak), while downregulation in mRNA genes that respond to peripheral blood mononuclear cells (PBMCs) has been shown in sports-related concussions [11].

Similarly, microRNAs (miRNAs) can influence biological processes after brain injury and may be useful in monitoring progression post-mTBI [12]. Certain biomarkers including miR-92a and miR-16 have been shown to play a role in inflammation and have been identified as elevated after an immediate mTBI, such as the elevation of IL-6 [12]. miRNAs are found at peak levels within the acute phase (two days), similar to IL-6, and begin to decrease when evaluated at the subacute phase (day 6 and day 13) [12]. miR-27a and miR-221 have been shown to decrease after a concussion in the subacute stage. It has also been shown that when levels of miR-221 decrease, it is an indication that a brain disruption is present. miR-221 is important in differentiation, cell proliferation, motility, and survival [12]. miR-27a also protects from neuronal apoptosis and regulates inflammation and the blood-brain barrier, a decrease in the level of miR-27a is present in brain injury [12].

Another potential biomarker of brain injury is ubiquitin C-terminal hydrolase-L1 (UCH-L1), which is present in the neurons and can help assess neuronal damage. In addition, glial fibrillary acidic protein (GFAP), a marker for glial damage, mainly involving brain lesions, and S100 calcium-binding protein beta (S100B), can serve as a marker for astrocyte damage. These markers have all been found elevated after a brain injury such as a mTBI [10]. Symptoms were elevated the most at the six-hour mark in the acute phase, in which both UCH-L1 and S100B were also at their peak (similar to interleukins and microRNAs), waning nearing the 48-hour mark [10]. mTBI athletes have demonstrated significantly elevated levels of UCH-L1 and S100B, but no change in GFAP, which is important as these markers may hold diagnostic value. Biomarkers have the potential to understand disease progression, recovery, and response to treatment. Continuous elevation of these markers may be a sign that brain injury and inflammation are likely present, as these markers are used to assess neuronal damage [4]. Additional research is warranted to address the limitations associated with the utilization of biomarkers for diagnosing concussions, including the evaluation of cost-effectiveness and the time required to obtain test results as concussions must be diagnosed in a timely manner. Furthermore, sample size, comorbidities, and demographic characteristics (i.e., age, gender, etc.) are considerations for future studies on this matter.

Short-Term Changes and Fatigue Levels After a Concussion

It has been shown that repeated history of concussions has a greater impact on changes in the brain. Like football, rugby is another sport that has been shown to have a high prevalence of concussions and causes long-term brain effects such as cognitive dysfunction [6]. Decreased cerebral blood flow may contribute to this cognitive dysfunction since there is a change in ion fluxes, neurotransmission, and impact on autoregulation [6]. Similarly, when it comes to short-term working memory, there is a significant negative impact following multiple concussions [6]. Post-concussion syndrome is common in almost all concussion cases and consists of poor short-term memory, headaches, and nausea. When an athlete succumbs to multiple concussions, post-concussion syndrome is heightened and there is greater impaired memory and attention [6]. 

An important consideration when evaluating concussions in athletes is baseline fatigue prior to athletes experiencing a concussion. Athlete fatigue may affect concussion assessment results since athletes typically hold rigorous training schedules, which often lead to sleep deprivation and subsequently, fatigue [13]. It is important to assess baseline[RJ1] fatigue prior to the concussion as well as reevaluate fatigue level after a concussion because both fatigue and concussions can cause cognitive dysfunction [13]. Fatigue is generally reported through a self-reported fatigue scale. The fatigue scale ranges from 1 to 100, with 1 being completely exhausted and 100 being fully awake and alert. Future studies should consider comparing reaction time and fatigue levels in athletes without a history of concussion to athletes with a history of concussion to assess the impact of fatigue levels in determining concussion severity.

Long-term effects of concussions

Persistent Concussion Symptoms

The long-term effects of sports-related concussions have been widely researched and have been found to affect all systems of the human body. In the brain itself, recent studies have shown that even after the symptoms of the affected athlete have resolved, changes to brain function persist. Through techniques measuring blood flow, metabolism, and glucose utilization in the brain, researchers have discovered that significant brain alterations may continue past the point when the athlete may appear to be symptom-free [5]. In addition, changes to the white matter of the brain may be present even after the patient no longer has symptoms. These findings may indicate that players who return to contact sports too soon may be at risk for additional physical injury [5]. According to a study by Govindarajan et al., there was a strong association between the number of previous concussions and future injuries, and a slower recovery was seen in athletes with multiple prior concussions [9]. This exposes them to further debilitating trauma if injured again. 

Academic Difficulty and Cognitive Deficits

The trauma that athletes are susceptible to is not just physical but can manifest in cognitive deficits as well. Many student-athletes are at risk of experiencing academic difficulty because of acute and prior concussions. Concussions have been shown to increase sensitivity to light and cause headaches and difficulty concentrating, which may lead to academic repercussions [7]. College student-athletes may have mandatory sessions such as team meetings, student organization meetings, study halls, and other events that may contribute to their academic standing. This may affect eligibility for scholarships or post-graduation opportunities. Without a guided return to learn protocol, athletes risk harming neural processing regions of the brain [7]. There is a strong call for accommodations and support for student-athletes healing from brain trauma as improperly healed or unhealed concussions can have disastrous effects on the cognitive functioning of the patient in the future.

In addition to significant cognitive deficits, depression, and other mental health complications have been identified in those who have been affected by head trauma. Recent studies examining autopsied specimens from retired athletes who have committed suicide found pathological markers present, which are consistent with those found in patients with chronic traumatic encephalopathy (CTE) [8]. CTE is distinguished by tau-immunoreactive neurofibrillary tangles and aggregation of tau-immunoreactive astrocytes without accumulation of β-amyloid seen in Alzheimer's disease. These markers were seen in the subjects, in addition to TAR DNA-binding 43-kDa proteinopathy in the frontal and temporal cortical regions and diencephalon. A few subjects also displayed a motoneuron disease characterized by weakness and TAR DNA-binding 43-kDa inclusions in the spinal cord [8]. In addition, considerable differences in white matter were seen when comparing depressed retired athletes and the control group. Regional blood flow differences were also associated with diminished cognitive performance [8]. As demonstrated by the referenced study, post-traumatic changes in the brain can manifest as depression and other mental health issues.

Gut Microbiome Changes

While concussion symptoms mostly affect the brain and cognitive processes, alterations to the gut microbiome have been seen after sports-related concussions in college athletes. Small changes in gut microbiota can significantly affect performance in athletics in addition to overall gut health. In one study, 16S rRNA sequencing was performed of the gut microbiome and it uncovered a reduction in two bacterial species, *Eubacterium rectale* and *Anaerostipes hadrus*, in the gut after a diagnosed concussion, while they were seen in abundance in athletes who did not suffer a diagnosed concussion. Both bacterial species are major butyrate producers with anti-inflammatory properties with *E. rectale* being associated with insulin metabolism [14]. A reduction in both species is correlated with pro-inflammatory processes and can cause further brain inflammation post-concussion. Concussion has been shown to trigger delayed changes in the colon and any bacterial infections in the gastrointestinal tract can increase post-concussion brain inflammation and degeneration of the brain in mice [14]. The study of these microbiome changes of the gastrointestinal tract has been focused only on male collegiate football players; however, post-concussive trauma can affect individuals of any gender, age, and sport.

Gender and Age Differences

Different genders and ages have been shown to exhibit varying cognitive and bodily changes post-trauma. One study demonstrated that female patients were more likely to present with a concussion from a sport and endure discomfort from the concussion, while males were more likely to experience amnesia, confusion, and loss of consciousness after a concussion [15]. Both males and females demonstrated a decrease in cognitive testing, while visual memory was more affected in females than in males [15]. With these findings, it has been shown that there are gender differences in post-concussion symptoms.

Age has also been shown to be a contributing factor to post-concussive symptoms. Older student-athletes who had experienced concussions were able to participate in academics for a longer period without experiencing concussion symptoms, while younger students showed less tolerance [16]. The study conducted by Covassin et al. focused on sex and age differences in post-concussion symptoms, neurocognitive testing, and postural stability. All 296 athletes participated in the Immediate Post-Concussion Assessment and Cognitive Test (ImPACT) and Post-Concussion Symptom Scale (PCSS) at baseline prior to experiencing a concussion. The ImPACT and PCSS scales were repeated after day 2, day 7, and day 14 after experiencing a concussion. The balance error scoring system (BESS) scale was repeated on day 1, day 2, and day 3 after a concussion [16].

It was noted in the study by Covassin et al. that younger athletes who experienced a concussion displayed considerable deficits in cognitive functioning and reaction time [16]. Younger athletes, between the ages of 10 and 17, have been shown to display irregularities in emotional and somatic well-being up to at least five weeks after trauma. The younger students in Covassin et al.'s study were much less tolerant of engaging in academic tasks after a concussion than older students [16]. Younger brains exposed to injury encounter stimuli differently than older patients with fully developed brains. Younger patients exposed to brain trauma exhibit increased impulsive response, in addition to psychomotor speed and visual-spatial skills impairment compared to their older counterparts [16]. It was found that high school students have more difficulty in math post-concussion, while college students have more difficulty in reading and computer use [16]. In addition, high school athletes displayed worse results on verbal and visual memory tests when compared to college athletes after brain trauma. Furthermore, the BESS assessed the static postural stability in athletes’ post-concussion [16]. While high school male athletes performed worse than their college counterparts on the BESS, college female athletes scored worse than their high school counterparts [16]. While there are discrepancies in what areas of academics are more difficult for different ages, concussions trigger negative symptoms and reduce academic performance regardless of age or gender.

Gait Change and Increased Risk for Lower Extremity Injury

Concussive events have been shown to negatively impact motor functions and psychomotor functions. Headache and dizziness caused by brain trauma can further contribute to postural instability and gait change. These changes are often assessed by the Rhomberg test and the BESS test. According to one study, balance deficits can be seen three to five days after injury, while others have reported physical instability at 3.7 years after a concussion [17]. These gait changes include slower walking speeds, shorter stride length, and shorter time in single-leg stance to preserve stability and decrease the possibility of further injury (i.e., fall). In addition, these gait changes were further evident as the patient simultaneously completed another cognitive task [17]. In athletes with two or more diagnosed concussions, it was found that they walked with a smaller stride length during dual-task gait examination than the control group [18]. The dual-task examination included walking while performing a cognitive test [18].

In addition to shorter strides and slower gait, concussion can also lead to an increased risk of lower extremity injury in athletes 90 days post-concussion. With a sample size of 153 athletes, Jildeh et al. demonstrated that 25.7% of individuals sustained a lower extremity injury within 90 days of their previous concussion [4]. The risk of developing an acute lower extremity musculoskeletal injury within 90 days of the previous concussion was 4.69 times greater than the control group. The lower extremity injuries sustained included ligament tears and sprains. Injuries were also found at the knee and thigh, ankle and hip, and groin [4]. In addition, lower extremity stiffness has also been seen in athletes after concussion. In one study, concussed athletes exhibited increased stiffness in the lower extremities compared to the uninjured group [19]. The concussed athletes also demonstrated knee stiffness and decreased knee angular excursion. Further research uncovered decreased quadriceps strength in collegiate football athletes as well. It is hypothesized that these altered movement patterns may be due to a reduced brain capacity for motor planning in athletes post concussion. This may include increased intracortical inhibition, decreased metabolic activity, and decreased intracortical excitability [19]. It would be important for future studies to focus on correlating concussions with specific lower extremity injuries.

## Conclusions

Concussions are a type of mTBI that can lead to severe long-term complications. They are very prevalent in high-impact sports such as football and boxing. Symptoms include dizziness, nausea, vomiting, headaches, and photosensitivity, which are common to many other injuries and illnesses. This makes diagnosis challenging. Currently, the diagnosis of concussion is based on physical evaluation, and early diagnosis and management are crucial for recovery. Researchers have used neuroimaging techniques such as DTI and DKI to evaluate the structural and chemical alterations in the brain. In addition, cortical thickness has been used to evaluate the effect of inflammation in the brain post concussion. While concussions have a significant impact on brain structure, they can also cause impairments to an athlete’s functioning. Recent research has concentrated on short-term changes and fatigue levels to understand the progression of concussions in athletes. Furthermore, repeated history of concussions has been shown to have a greater impact on the brain, including decreased cerebral blood flow and impaired short-term memory. These findings determine how imperative it is to immediately treat concussions to combat deleterious changes to the brain. A potential diagnostic aid is that of biomarkers, such as IL-6, as they can monitor the progression of concussions and the efficacy of treatment methods used.

Concussions have been found to affect all systems of the human body including cognitive function, mental health, and the gut microbiome. Repeated brain insults have been associated with a slower long-term recovery. They have also been shown to increase sensitivity to light and cause headaches and difficulty concentrating which may affect academic studies. Depression and other mental health complications have been identified in those affected by head trauma. In addition, alterations to the gut microbiome have been seen after sports-related concussions in college athletes, with significant differences between males and females. Similarly, age is seen as a contributing factor to post-concussive symptoms, with younger athletes displaying more cognitive deficits and less tolerance to participation in academic tasks after a concussion. Concussions have a significant impact on various body systems, emphasizing the importance of effectively addressing the symptoms of concussions in athletes to further allow complete recovery and reduce the likelihood of future concussions.
